# EBV DNA methylation profiles and its application in distinguishing nasopharyngeal carcinoma and nasal NK/T-cell lymphoma

**DOI:** 10.1186/s13148-024-01624-y

**Published:** 2024-01-11

**Authors:** Cao-Li Tang, Xi-Zhao Li, Ting Zhou, Chang-Mi Deng, Cheng-Tao Jiang, Yu-Meng Zhang, Ying Liao, Tong-Min Wang, Yong-Qiao He, Wen-Qiong Xue, Wei-Hua Jia, Xiao-Hui Zheng

**Affiliations:** 1 State Key Laboratory of Oncology in South China, Guangdong Key Laboratory of Nasopharyngeal Carcinoma Diagnosis and Therapy, Guangdong Provincial Clinical Research Center for Cancer, Sun Yat-sen University Cancer Center, 651 Dongfeng East Road, Guangzhou, Guangdong 510060 People’s Republic of China; 2 School of Public Health, Sun Yat-sen University, Guangzhou, 510080 China

**Keywords:** EBV, EBV DNA methylation, EBV-associated tumor, NPC, Nasal NKTCL

## Abstract

**Background:**

As an oncovirus, EBV is associated with multiple cancers, including solid tumors and hematological malignancies. EBV methylation plays an important role in regulating tumor occurrence. However, the EBV methylation profiles in EBV-associated tumor tissues are poorly understood.

**Results:**

In this study, EBV methylation capture sequencing was conducted in several different tumor tissue samples, including NPC, EBVaGC, lung LELC and parotid LELC. Besides, EBV capture sequencing and following qMSP were performed on nasopharyngeal brushing samples from NPC and nasal NKTCL patients. Our results showed that the EBV genome among different types of tumors displayed specific methylation patterns. Among the four types of tumors from epithelial origin (NPC, EBVaGC, lung LELC and parotid LELC), the most significant differences were found between EBVaGC and the others. For example, in EBVaGC, all CpG sites within 1,44,189–1,45,136 bp of the EBV genome sequence on gene RPMS1 were hyper-methylated compared to the others. Differently, significant differences of EBV CpG sites, particularly those located on gene BILF2, were observed between NPC and nasal NKTCL patients in nasopharyngeal brushing samples. Further, the methylated level of BILF2 was further detected using qMSP, and a diagnostic model distinguishing NPC and nasal NKTCL was established. The AUC of the model was 0.9801 (95% CI 0.9524–1.0000), with the sensitivity and specificity of 98.81% (95% CI 93.63–99.94%) and 76.92% (95% CI 49.74–91.82%), respectively.

**Conclusions:**

Our study reveals more clues for further understanding the pathogenesis of EBV, and provides a possibility for distinguishing EBV-related tumor by detecting specific EBV CpG sites.

**Supplementary Information:**

The online version contains supplementary material available at 10.1186/s13148-024-01624-y.

## Background

Epstein–Barr virus (EBV) is a ubiquitous human virus that affects almost 95% of people worldwide. After primary infection, the virus usually establishes life-long latency in memory B cells [1]. Though the infection is common and generally asymptomatic, EBV has been associated with multiple malignancies, including solid tumors [such as nasopharyngeal carcinoma (NPC) [2], EBV-associated gastric cancer (EBVaGC) [3], lymphoepithelioma-like carcinoma (LELC) [4], etc.] and hematological malignancies [including Burkitt's lymphoma (BL) [5], non-Hodgkin lymphoma (NHL) [6], Hodgkin's lymphoma (HL) [7], etc.].

Currently, it is not very clear how the virus affects the development of tumor, but there have been studies that provide some clues. DNA methylation are common epigenetic alterations observed in viral oncogenesis [8]. Taking EBV in NPC as an example, the LMP1 protein from EBV genome can interfere with the activity of DNMTs enzyme, leading to the methylation modification of the promoter regions of genes such as E-cadherin [9], RAR-β2 [10], RASSF1A [11, 12] and CDKN2A [11, 12], thereby affecting their expression. Therefore, it can be inferred that CpG sites methylation plays a role in the pathogenesis of NPC. Methylation of the EBV genome also plays an important role in regulating its own lifecycle. The complete EBV particle is epigenetic-naïve [13]. However, it uses the DNMTs of host cells to carry out methylation modifications after infecting the host cell [13]. The methylation status of promoter regions on the EBV genome varies during different lytic cycles, thereby regulating the expression of different genes [14]. In most infected cells, the EBV genome is in an epigenetically suppressed state, thereby resulting in only a small amount of EBV protein expression, and enabling it to evade immune surveillance in the host and persist in a stable latent state within the cells [15, 16]. Therefore, it can be inferred that EBV DNA appears methylated in most infected cells [17]. However, the EBV methylation profiles in EBV-associated tumor have been not well understood, especially in tumors originated from the same cell type, such as epithelial cells. Further methylation analysis will inform more etiologies of EBV-associated diseases.

In previous studies including ours, EBV DNA showed significant difference in specific CpG sites by capture sequencing in plasma and saliva samples between NPC patients and non-NPC controls, showing potential in NPC's diagnosis [18, 19]. In addition, we have previously developed a nasopharyngeal brush sampling method, and examined several biomarkers, including methylation status of EBV DNA C promoter in nasopharyngeal brushing samples of NPC cases and controls, and suggested they could serve as a valuable method in the diagnosis of NPC [20–24]. Nasal NK/T-cell lymphoma (NKTCL), although a rare tumor with similar disease site and symptoms to NPC [25], usually leads to interference. EBV methylation analysis may provide the possibility for further distinguishing NPC and nasal NKTCL.

In this study, EBV methylation capture sequencing was firstly conducted in several different tumor tissues all originated from epithelial cells, including NPC, EBVaGC, lung LELC and parotid LELC. Secondly, EBV capture sequencing and following qMSP were performed on nasopharyngeal brushing samples from NPC and nasal NKTCL patients. The results showed that EBV genome among different types of tumors displayed specific methylation patterns. Our study reveals more clues for further understanding the pathogenesis of EBV, and provides a possibility for distinguishing EBV-related tumor by detecting specific EBV CpG sites.

## Methods

### Tissue samples

Fresh-frozen tumor tissue samples were collected from 40 EBV-positive cancer patients in Sun Yat-sen University cancer center, including ten NPC tissues, ten EBVaGC tissues, ten lung LELC tissues, and ten parotid LELC tissues, the clinicopathologic characteristics of which were summarized in Additional file 1: Table S1. These samples were cryopreserved within 30 min in liquid nitrogen after surgical resection and stored in − 80 °C until DNA extraction.

### Nasopharyngeal brushing samples

The sampling method used was blind brushing, which means sampling without guidance of a nasal endoscope as previously described [20, 22, 24]. In brief, prior to biopsy sampling, trained personnel inserted a nasopharyngeal brush (Copan Diagnostics, Murrieta, CA) deeply into the patient's nasopharynx and rotated it several times against the nasopharyngeal epithelium to collect the sample. After sampling, the brush tip (1.5 cm) was immediately cut and placed in 1 mL of RNAlater (Invitrogen, Carlsbad, CA) and finally stored at − 80 °C until DNA extraction. A total of 109 NPC and 18 nasal NKTCL patients’ samples were collected at Sun Yat-sen University cancer center, with 6 NPC and 3 nasal NKTCL patient samples used for bisulfite sequencing and the remaining samples used for qMSP detection. The clinicopathologic characteristics of these patients were summarized in Additional file 1: Table S1.

### DNA extraction, EBV genome enrichment, and bisulfite sequencing

DNA was extracted from the samples using Chemagic Star workstation (Hamilton, Perkin Elmer, Waltham, USA) as described in our previous study [20, 22, 24]. The pool tissue DNA libraries were constructed from each ten patients of the same tumor and subjected to EBV-targeted bisulfite sequencing. Briefly, the DNA pools were first subjected to random fragmentation, end repair, A-tailing, and methyl-adaptors ligation. Hybridization capture (VariantBaits™ Target Enrichment Library Prep Kit, LCBio Tech, Hangzhou, China) was then performed using the EBV-targeting single-stranded DNA probes (Integrated DNA Technologies, Coralville, IA, USA) to enrich the EBV genomes. The captured DNA was then subjected to bisulfite conversion (EZ DNA Methylation Gold Kit, Zymo Research, Irvine, CA, USA) and PCR amplification (SureSelect Methyl-Seq PCR Kit, Agilent Technologies, Santa Clara, CA, USA). The preprocessed libraries were sequenced using the NovaSeq 6000 platform (Illumina, San Diego, CA, USA).

### Quantitative polymerase chain reaction (qPCR) analysis

The EBV DNA loads in samples were measured by qPCR, with amplification primers and a dual-labeled hybridization probe targeting the BamHI-W region. Each reaction volume of 8μL containing 4μL PCR master mix, 1μL primers, 0.2μL probe, 0.8μL nuclease-free water and 2μL DNA. PCR reaction was performed in LightCycler 480 (Roche Diagnostics, Basel, Switzerland) under the following conditions: 5 min at 95 °C, and then 45 cycles of 95 °C for 15 s, 60 °C for 30 s and 72 °C for 15 s and finally 72 °C cooling for 5 min. Plasmid DNA containing the target region in serial dilution (10^3^, 10^4^, 10^5^, 10^6^ and 10^7^ copies/μL) was used to establish the standard curve for absolute quantification. The β-globin DNA was detected to evaluate the quality of blind brushing samples. And the samples with an amplification Ct value < 30 were considered valid. The detection method of β-globin was similar to the method used for EBV DNA load detection. Besides, the quantitative methylation-specific PCR (qMSP) method was used to detect the methylation status of the target region on gene BILF2 in the nasopharyngeal brushing samples. Specifically, bisulfite-converted DNA was used as a template for amplification, with each reaction volume of 20 μL containing 10 μL PCR master mix, 1.5 μL primers, 0.8 μL methylated probe, 0.8 μL unmethylated probe, 5.9 μL water and 1μL DNA template. PCR condition was set up as follows: 5 min at 95 °C, and then 45 cycles of 95 °C for 15 s, 58 °C for 60 s and finally 40 °C cooling for 30 s. The CtM for methylated probe and CtU for unmethylated probe can be obtained. Two plasmids containing the completely methylated and completely unmethylated sequence respectively were constructed for quality assessment of the methylation detection. According to the result, there was no evidence of non-specific amplification within the same reaction. The amplification efficiencies of methylated and unmethylated products were 90.12% and 100.15%, respectively (Additional file 1: Table S2; Fig S1). If CtM or CtU was missing, a value of 45 cycles was used to fill in. If both values cannot be obtained, the sample will be excluded. The relative methylation level of the BILF2 gene was reflected using − ΔCt (cycle threshold) and compared between groups where ΔCt = CtM − CtU. All primers and probe sequences were shown in Additional file 1: Table S3.

### Bioinformatic and statistical analysis

Sequenced reads were in silico aligned to the reference bisulfite-converted EBV genome after filtering for low-quality loci with CpG sites count < 10. The methylation density of each CpG sites was calculated as the fraction of unconverted cytosines (methylated) over the sum of unconverted cytosines (methylated) and converted thymine (unmethylated) present in its total depth. Methy-Pipe [26] software was used to complete bioinformatic analysis. Differences in methylation between two groups were tested using the Mann–Whitney U test. Receiver operating characteristic (ROC) curve was generated and area under the curve (AUC) was calculated to evaluate the diagnostic performance of marker. All statistical tests were two-sided and set *α* equal to 0.05. All statistical analyses were performed using R software, version 4.1.2 (http://www.r-project.org).

## Results

### EBV genome methylation profiles in EBV-associated tumor tissues from epithelial cells

A total of 6260 CpG sites from EBV strains were identified in NPC tissues, and 3602 in EBVaGC, 7057 in lung LELC and 6736 in parotid LELC tissues, among which, a total of 3497 CpG sites were detected in all four libraries.

**Fig. 1:**
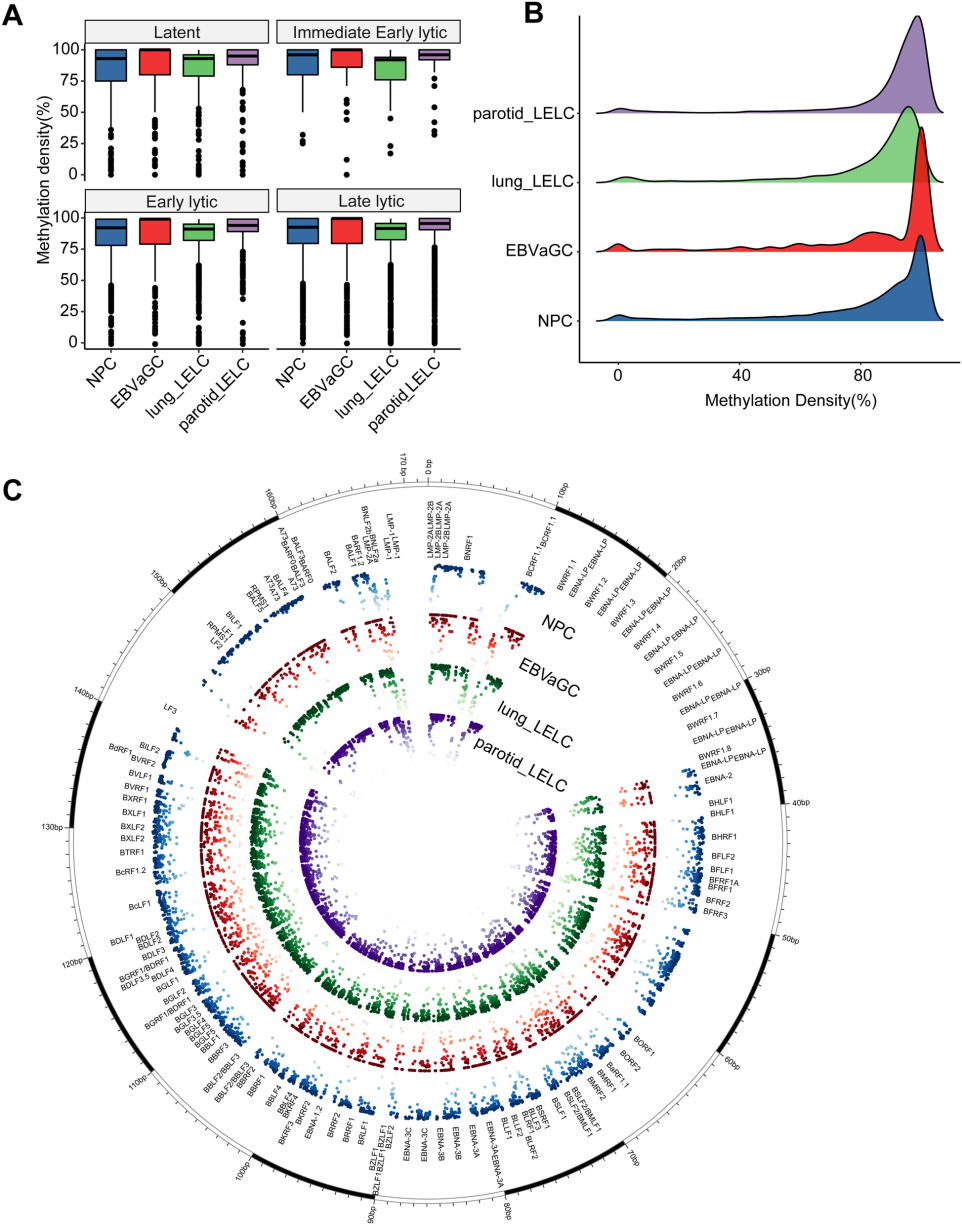
Distribution of EBV CpG sites methylation levels in four tumor tissue samples. **A** Box plots of the average methylation density of CpG sites annotated on different phases genes. **B** Methylation density plot of EBV CpG sites in NPC, EBVaGC, lung LELC and parotid LELC tissue samples. The higher the ridge, the more CpG sites at that methylation level. **C** EBV genome circos plot. The inner circle was a scatter plot of methylation density of EBV sites in each tumor tissue, where different colors represented different types of cancer, and darker colors indicated higher levels of methylation

The average methylation density of the 3497 CpG sites was highest in parotid LELC (87.93%), followed by EBVaGC (83.18%), lung LELC (83.04%) and NPC (82.35%). To further compare the methylation of different functional sites, we annotated all 3497 CpG sites to EBV genes and classified them into latent, immediate early lytic, early lytic genes and late lytic genes according to the period of gene expression, and the average methylation density of each classification was calculated (Fig. 1A). Most of the loci on different phase genes were hyper-methylated and the methylation density was comparable in the four kinds of tumors. The methylation distribution plot and circos plot (Fig. 1B–C) also confirmed that the vast majority of EBV loci were hyper-methylated in these four types of tumor tissues, with few loci showing hypo-methylation.

**Fig. 2:**
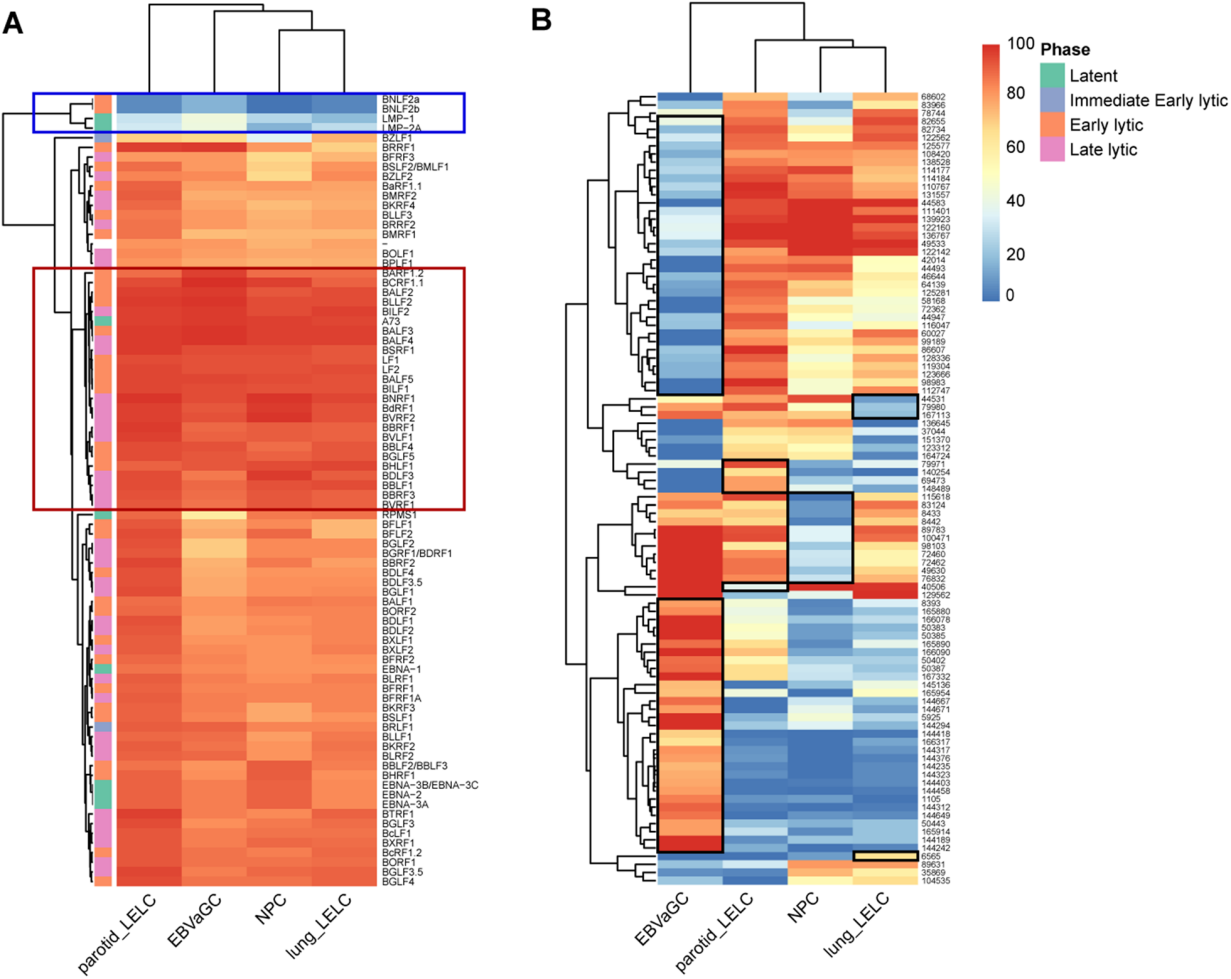
Heatmap of EBV methylation density in four tumor tissue samples. **A** Methylation density heatmap of 81 EBV genes annotated with 3497 CpG loci in NPC, EBVaGC, lung LELC and parotid LELC tissues. The redder the color, the higher the methylation density, and the bluer the lower the methylation density. The genes within the red boxed area were relatively hyper-methylated; while, the genes within the blue boxed area were relatively hypo-methylated. **B** Methylation density heatmap of the most variable loci among these tumor tissues. In the heatmap, the black box indicated the loci with significantly higher or lower methylation levels in the corresponding tumor tissue, compared to other tumor tissues

In order to investigate the methylation patterns of EBV genes in these tumor tissues, we annotated the 3497 captured CpG sites, which were captured in all four cancers, to the EBV genome and generated a heatmap based on the methylation density of each gene. As shown in Fig. 2A, the loci on two early lytic phase genes (BNLF2a and BNLF2b) as well as two latent phase genes (LMP1 and LMP2A) were found to be hypo-methylated in all four types of cancer tissues. In contrast, many other early lytic phase genes (such as LF1, LF2, BALFs, etc.) and some late lytic phase genes (such as BNRF1, BVRF2, BVLF1, BSRF1, etc.) exhibited a hyper-methylated state. These results suggested a similar methylation pattern of EBV in different tumor tissues.

However, differences in methylation sites still existed among different types of cancer. The methylation densities of these 3497 CpG sites were compared among different cancer groups, and the loci with the most variable methylation densities among these four kinds of tumor tissue (standard deviation > 30) were selected for plotting a heatmap (Fig. 2B). The heatmap results revealed that each tumor had CpG sites with unique patterns of higher or lower methylation density compared to the other tumors, and the differences between EBVaGC and other cancer types were significant. It was noteworthy that in comparison with other tumors, EBVaGC sample was highly methylated on all adjacent CpG sites within 1,44,189–1,45,136 bp of the EBV sequence, which was located on gene RPMS1 (Additional file 1: Figure S2). In addition, parotid LELC sample showed specific hyper-methylation at CpG site 69,473 bp, 79,971 bp, 140,254 bp and 1,48,489 bp. The first two were located at the overlap region of genes EBNA1, EBNA3A, and EBNA3B/3C; while, the latter two were located on RPMS1. Lung LELC sample showed specific hypo-methylation at 44,531 bp, 79,980 bp and 1,67,113 bp and NPC sample exhibited hypo-methylation at 8433 bp, 8442 bp, 49,630 bp, 72,460 bp, 72,462 bp, 76,832 bp, 83,124 bp, 89,783 bp, 98,103 bp, 1,00,471 bp and 1,15,618 bp. Most of these sites located on the overlap region of genes EBNA1, EBNA3A, and EBNA3B/3C.

The above results indicated that the methylation density of CpG sites on the EBV genome was not entirely consistent across different tumor tissues, and each type of tumor had specific methylation sites.

### EBV genome methylation profiles in NPC and nasal NKTCL

**Fig. 3:**
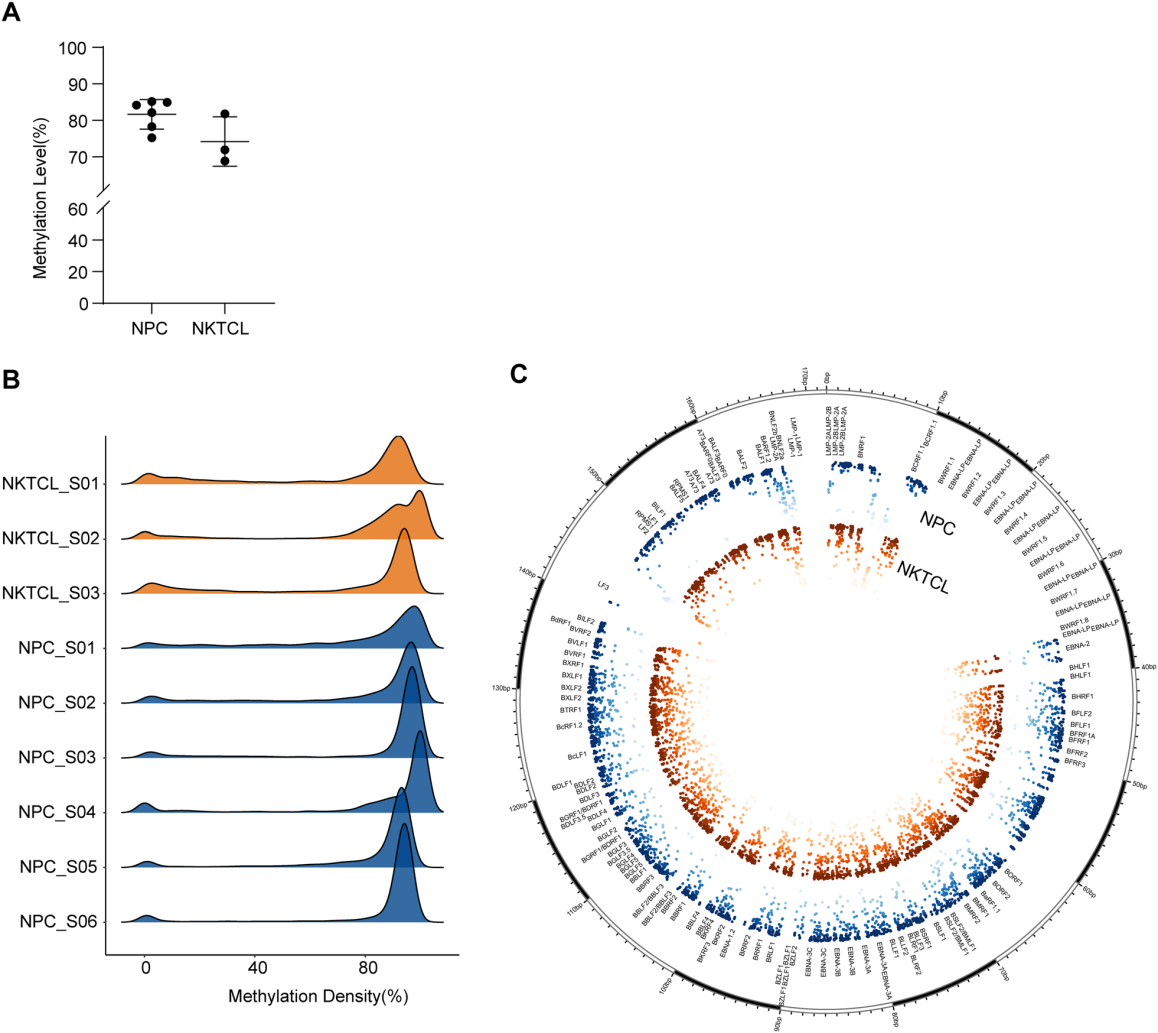
Distribution of EBV CpG sites methylation levels in NPC and nasal NKTCL samples. **A** The comparison of average methylation levels between NPC and nasal NKTCL nasopharyngeal brushing samples. **B** Methylation density plot of EBV CpG sites in NPC and nasal NKTCL nasopharyngeal brushing samples. The higher the ridge, the more CpG sites at that methylation level. **C** EBV genome circos plot. The inner circle was a scatter plot of methylation density of EBV sites in each nasopharyngeal brushing sample group, where different colors represented different types of cancer, and darker colors indicated higher levels of methylation

We further performed EBV genome capture sequencing on nasopharyngeal brushing samples from six NPC patients and three nasal NKTCL patients. A total of 3683 CpG sites were detected in all samples and included in downstream analysis. The average level of EBV methylation was high in both sample groups, but slightly higher in NPC compared to nasal NKTCL (Fig. 3).

**Fig. 4:**
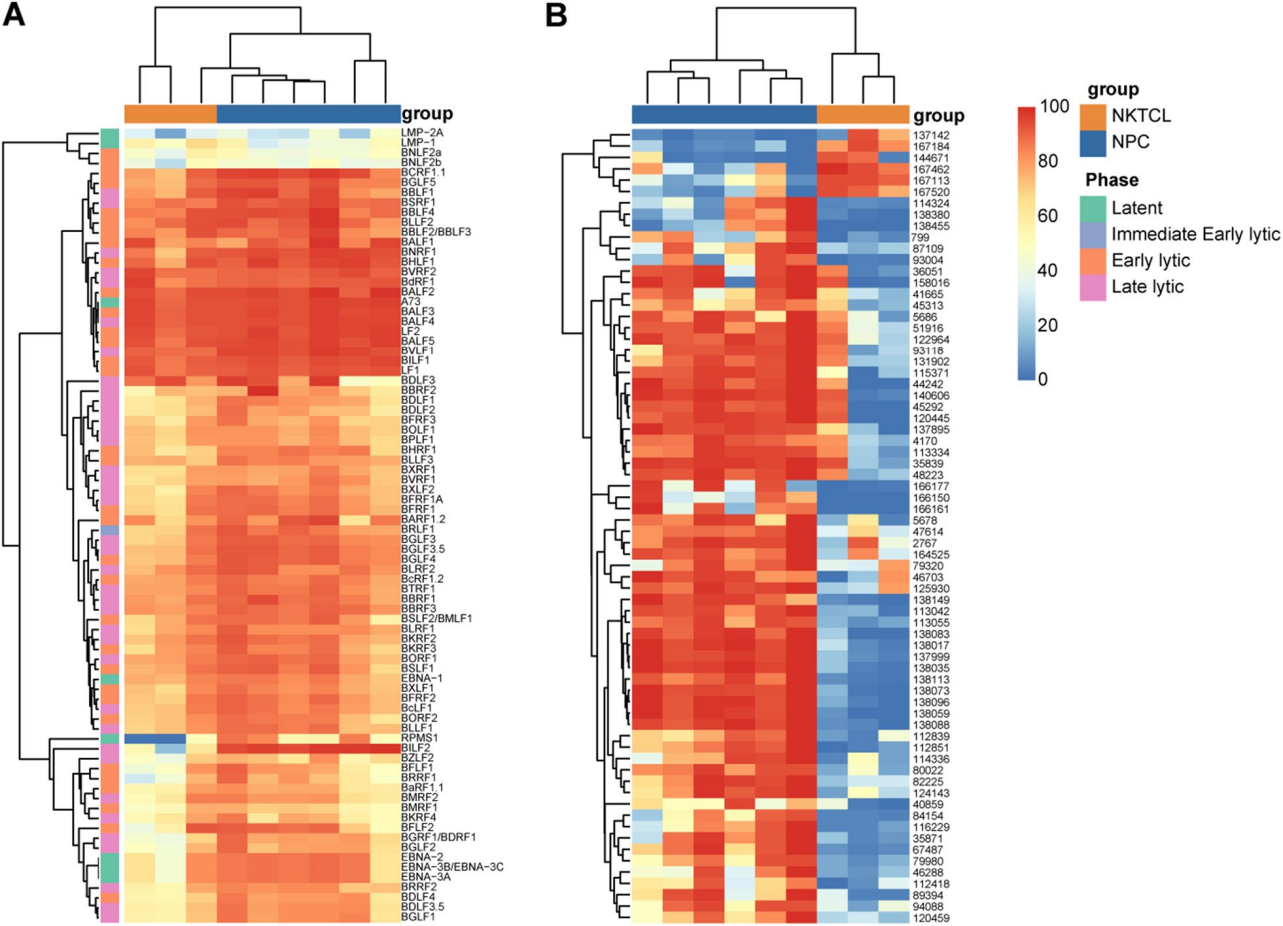
Heatmap of EBV methylation density in NPC and nasal NKTCL nasopharyngeal brushing samples. **A** Methylation density heatmap of 80 EBV genes annotated with 3683 CpG loci in NPC and nasal NKTCL nasopharyngeal brushing samples. The redder the color, the higher the methylation density, and the bluer the lower the methylation density. **B** Methylation density heatmap of the most variable loci among these nasopharyngeal brushing samples

Additionally, we annotated 3683 CpG sites to the EBV genome and obtained 80 genes methylation density. As shown in Fig. 4A, the loci on genes BNLF2a, BNLF2b, LMP1 and LMP2A were found to be hypo-methylated in all nasopharyngeal brushing samples; whereas, many early lytic phase and late lytic phase genes including LF1, LF2, BNRF1, and so on were hyper-methylated. Despite this, methylation differences persisted between the two groups, and nasopharyngeal brushing samples from different patients with the same cancer were almost clustered together in the cluster analysis. This suggested that the methylation profiles of the EBV genome were distinct between NPC and nasal NKTCL, and patients with the same cancer would share a similar methylation pattern. Among these 80 genes, thirteen genes showed statistically significant differences between NPC and nasal NKTCL (Table 1). The noteworthy point was that there was a significant difference in gene BILF2 between the two groups (Fig. 4A; Additional file 1: Fig. S3A).

**Table 1:** Differentially methylated (*P* < 0.05) EBV genes in NPC and nasal NKTCL nasopharyngeal brushing samples

EBV Gene	Median methylation level (%)		*P*^a^	Phase
	NPC	NKTCL		
BNRF1	92.85	85.82	0.024	Late lytic
BCRF1.1	93.74	78.90	0.048	Early lytic
BSRF1	90.52	84.34	0.048	Late lytic
BLLF3	81.57	74.31	0.024	Early lytic
BBRF3	87.62	80.59	0.048	Late lytic
BGRF1/BDRF1	78.17	45.12	0.048	Late lytic
BDLF3.5	81.56	58.08	0.048	Late lytic
BDLF4	80.04	54.83	0.048	Early lytic
BGLF1	81.33	56.83	0.048	Late lytic
BVLF1	94.31	87.54	0.024	Late lytic
BILF2	96.00	53.72	0.024	Late lytic
RPMS1	69.42	1.48	0.024	Latent
BILF1	92.81	90.16	0.048	Early lytic

^a^*P* value of the Wilcoxon test

Furthermore, we conducted Wilcoxon test on the differences in the methylation levels of each captured CpG sites between NPC and nasal NKTCL nasopharyngeal brushing samples and obtained 288 differentially methylated sites (*P* < 0.05). 70 specific CpG sites were found to exhibit significant differences between groups, with a median difference of methylation density ≥ 50%. A heatmap was generated using the methylation density of these sites (Fig. 4B). Most of the sites showed hyper-methylation in NPC samples, with the exception of six sites that were mainly annotated to latent gene LMP1 and exhibited hyper-methylation in nasal NKTCL samples. The largest number of hyper-methylated sites within the NPC samples was located on the BILF2 gene. To further investigate the methylation status of this gene in the two groups of samples, we compared the methylation density of all sites on this gene (Additional file 1: Fig. S3B). The sequence of the BILF2 gene contains a total of 27 CpG sites, all of which were successfully captured in all samples. The comparative results showed that the last ten adjacent CpG sites on this gene exhibited significant differences between the two groups: These sites were almost completely methylated in NPC samples; while, they were almost completely unmethylated in nasal NKTCL samples.

**Fig. 5:**
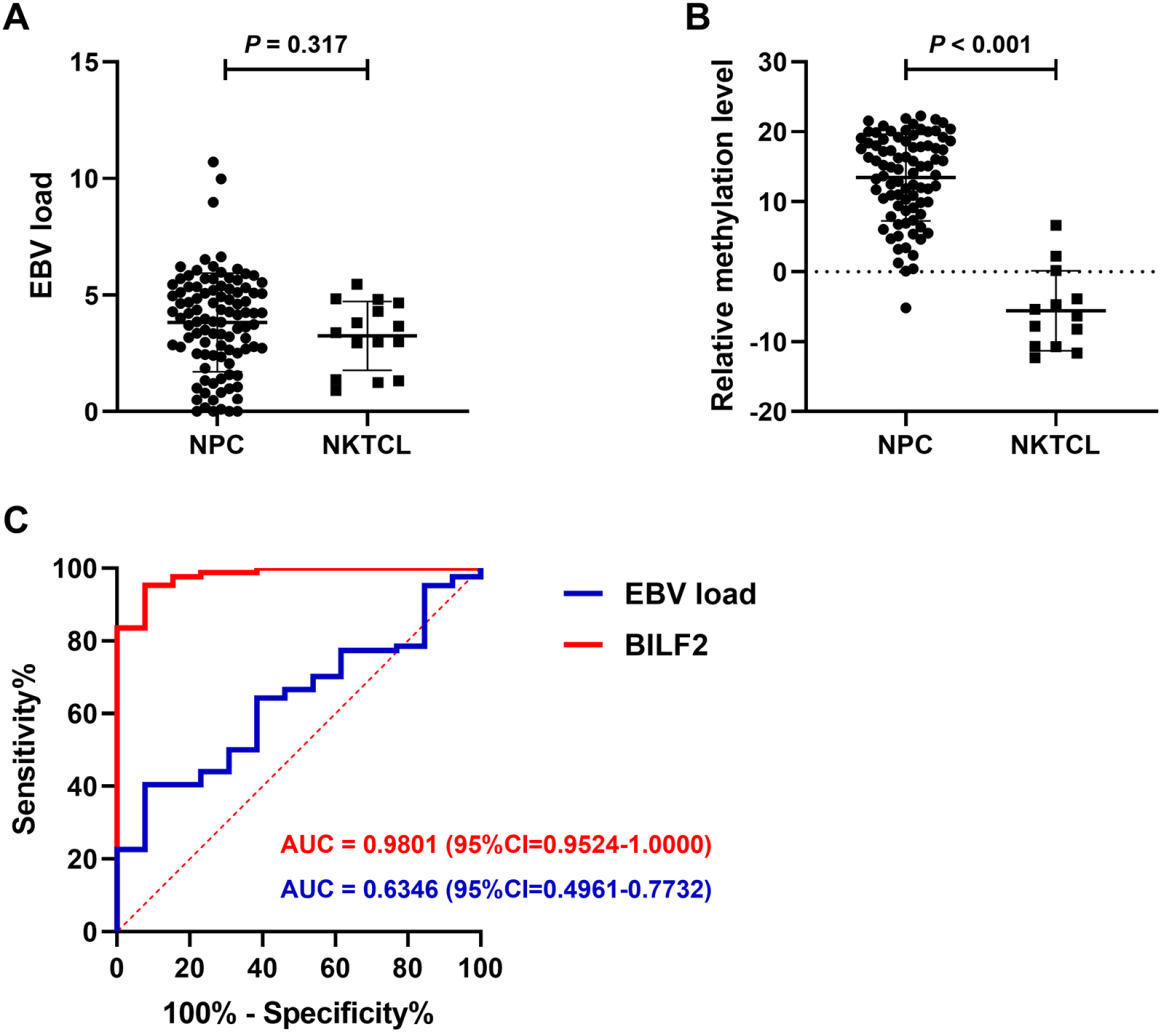
Methylation of BILF2 and EBV load detected by qPCR between NPC and nasal NKTCL samples. **A** Comparison of EBV load results between NPC and nasal NKTCL nasopharyngeal brushing samples. **B** Comparison of BILF2 qMSP results between NPC and nasal NKTCL nasopharyngeal brushing samples. **C** ROC curve for BILF2 qMSP and EBV load results

To validate these results, we collected nasopharyngeal brushing samples from 103 NPC patients and 15 nasal NKTCL patients, and performed qMSP detection. The β-globin DNA was detected to evaluate the sample quality before qMSP detection. Four NPC samples showed an amplification Ct value > 30 and were excluded. The copy number of the β-globin DNA per ngDNA exhibited no statistical difference between the two groups (Additional file 1: Fig. S4). In addition, we also tested EBV load in these two groups of samples and the results showed that there was no statistically significant difference between the two groups (Table 2; Fig. 5A). Regarding BILF2 detection, two nasal NKTCL samples and fifteen NPC samples that could not be detected due to low EBV load were also excluded. Based on the detection of methylated and unmethylated DNA products, the remaining samples could be divided into three types. Type one refers to samples that only produced methylated products (M), which means only the Ct value of M can be obtained. Type two refers to samples that only produced unmethylated products (U), which means only the Ct value of U can be obtained. Type three refers to samples that produced both types of products, which means both the Ct values of M and U can be obtained. The observation revealed a significant difference in the methylation category between the NPC and nasal NKTCL. In NPC samples, 68 out of 84 cases were classified as type one, while in nasal NKTCL samples, 10 out of 13 cases were classified as type two (Table 2; Fig. 5B). This indicated that most NPC samples were almost completely methylated within this region; while, they were almost completely unmethylated in nasal NKTCL, which was consistent with the capture sequencing results.

**Table 2:** Methylation of BILF2 and EBV load in the nasopharyngeal brushing samples detected by qPCR

Group	EBV load^a^			BILF2^b^				
	*N*	$\overline{x}$ ± *s*	*P*	N^c^			$\overline{x}$ ± *s*	*P*
				*M*	*U*	*M* + *U*		
NKTCL	15	3.83 ± 2.11	0.317	1	10	2	− 5.57 ± 5.72	< 0.001
NPC	99	3.26 ± 1.47		68	1	15	13.46 ± 6.23	

^a^The calculation method for EBV load indicators is log_10_(copy/μl + 1)

^b^The calculation method for BILF2 methylation indicators is –(CtM–CtU)

^c^Samples for which Ct values of both methylated and unmethylated products cannot be obtained are removed

Further, using the qMSP results, we constructed a model to distinguish NPC from nasal NKTCL by setting zero as the cutoff value (COV). The sensitivity and specificity of the model were 98.81% (95% CI 93.63–99.94%) and 76.92% (95% CI 49.74–91.82%), respectively, and the area under the ROC curve (AUC) was 0.9801 (95% CI 0.9524–1.0000). Thus, the model was able to effectively distinguish between the two groups. However, the two groups could not be well distinguished by EBV load. Although the specificity could reach 92.31% (95% CI 66.69–99.61%) when using the best cutoff value as COV, the sensitivity was only 40.48% (95% CI 30.62–51.17%) and the AUC was 0.6346 (95% CI 0.4961–0.7732), and the performance was inferior to the model constructed by BILF2 methylation index (Fig. 5C).

## Discussion

In this study, we first performed EBV methylation capture sequencing on several different tumor tissue samples both originated from epithelial cells, including NPC, EBVaGC, lung LELC and parotid LELC, and found specific methylation differences at certain sites, such as some CpG sites on gene RPMS1 in EBVaGC tissues. Further we conducted capture sequencing and qMSP on nasopharyngeal brushing samples from two different types of cell origin including NPC and nasal NKTCL, and found significant differences in methylation density at CpG sites on gene BILF2 between the two groups.

According to the results of EBV capture sequencing (Fig. 1A), the average methylation density was slightly higher in parotid LELC (87.93%); while, the average methylation density was similar in lung LELC (83.04%), EBVaGC (83.18%), and NPC (82.35%). We detected the expression and protein levels of DNMTs in the corresponding samples. The results showed that the differences in mRNA levels among the four types of tumor tissues were not statistically significant (*P* > 0.05) (Additional file 1: Fig. S5A). At the protein level, there was no statistically significant difference in the protein levels of DNMT3B among these groups. However, differences in the protein levels of DNMT1 and DNMT3A among the groups were statistically significant, primarily reflected in relatively higher expression in the parotid LELC samples (Additional file 1: Fig. S5B–C). Thus, the protein levels of DNMTs may partially explain the difference in the average methylation density of captured EBV CpG sites. Most of the captured CpG sites were found to be hyper-methylated (Fig. 1B–C); while, some CpG sites on certain genes were hypo-methylated, including BNLF2a, BNLF2b, LMP1 and LMP2A (Fig. 2A), consistent with the results of Fernandez’s study [27]. In their study, they sequenced 77 EBV transcription start sites from benign proliferating cells, tumor tissues and cells, as well as free virus DNA. Our study utilized capture sequencing across a comprehensive region of the EBV genome, thereby filling in the gaps of unexplored areas in previous research. EBV undergoes progressive demethylation during lytic amplification, resulting in epigenetically naïve viral particles [13]. Based on these results, it can be inferred that most EBV in tumor tissue exists in infected cells rather than as naked viruses released into the surrounding environment.

However, there were also specificities in each type of tumor with methylation patterns distinct from other tumors (Fig. 2B). For example, the most significant differences between EBVaGC and the other tumors were found especially at CpG sites within the 1,44,189 to 1,45,136 bp of the EBV sequence on RPMS1 gene (Additional file 1: Fig. S2). Similarly, the other three types of tumors also exhibited specific hyper- or hypo-methylation sites, reflecting the specificity of EBV genome methylation in different diseases. In a previous study [18], methylation analysis on plasma EBV DNA from patients with infectious mononucleosis (IM), NPC, and EBV-related lymphoma were performed and disease specificity of the EBV genome methylation were found. Here, we further performed the methylation detection in the primary tissue samples, which might provide more direct clues on EBV methylation and its role in tumor occurrence.

Among the cancer-specific methylated sites, many were located on gene RPMS1. RPMS1 is the sole member of the BamHI-A rightward transcripts (BARTs) family for which a complete complementary DNA has been identified [28]. Previous studies have found that single nucleotide polymorphisms (SNPs) of RPMS1 are related to tumor typing [28, 29], for example SNP 155391 G > A is associated with a high risk of NPC [30]. The expression products of RPMS1 are considered to have carcinogenic potential [31–33], and its expression has also been observed in various tumors [34–36]. However, there is currently no sufficient research evidence to reveal how the RPMS1 protein exerts its carcinogenic effect [34]. This study provides some clues from an epigenetic perspective. The specific hyper-methylation of EBVaGC and parotid LELC at different sites on the RPMS1 gene suggests that it may play different functions in the occurrence of different diseases but further research is needed to confirm this.

Nasal NKTCL, although a rare tumor, is similar to NPC in terms of the disease site and symptoms [25], often leading to diagnostic interference. As a common pathogenic factor [37, 38], EBV has similar mutation load [39] and hotspots [37] in these two tumors. Drawing on the findings of the first part of this work, it was observed that the methylation modification of the EBV genome varied across different diseases. Hence, it was hypothesized that such variation could be used to differentiate between these two cancers. To test this hypothesis, we provide a comparison of EBV patterns in NPC and nasal NKTCL from an epigenetic perspective. Through EBV methylation capture sequencing on nasopharyngeal brushing samples from NPC and nasal NKTCL patients (Fig. 3B–C; Fig. 4A), we found that most of the CpG sites in these two group samples were hyper-methylated; while, a small portion of hypo-methylated genes included LMP1, LMP2A, BNLF2a and BNLF2b, which was consistent with other tumor samples. Remarkably, significant differences in the methylation patterns of CpG sites, particularly those located on gene BILF2, were observed between EBV derived from nasopharyngeal brushing samples of NPC and nasal NKTCL patients (Additional file 1: Fig. S3). In the NPC samples, the CpG site on gene BILF2 was found to be fully methylated, while in the nasal NKTCL samples, it was completely unmethylated (Fig. 4A–B), reflecting a significant difference in the methylation modification of these sites between the two diseases. Furthermore, we validated this result using the qMSP method (Table 2; Fig. 5). This finding may suggest a differential role of EBV BILF2 gene in the development of these two cancers, but further research is needed to confirm this. Based on the methylation level detected by qMSP method, we established a discrimination diagnostic model. The AUC of the model reached 0.9801 (95% CI 0.9524–1.0000), with sensitivity and specificity of 98.81% (95% CI 93.63–99.94%) and 76.92% (95% CI 49.74–91.82%), respectively. The performance of this model was better than the model constructed using EBV load which was often used in the diagnosis and prognosis of EBV-associated diseases [40–42]. Nasopharyngeal brush sampling is a straightforward tissue sampling method that offers non-invasiveness, ease of performance, acceptability, and effectiveness [20, 22, 43]. Given these advantages, it is expected that this technique will gain widespread usage. Furthermore, qMSP detection of BILF2 gene sites in nasopharyngeal brushing samples has the potential to serve as an auxiliary diagnostic tool for discriminating between NPC and nasal NKTCL cases.

BILF2 is a gene expressed in the late lytic phase of EBV. It encodes a glycoprotein with an N-linked, gp78/55, and this membrane protein is detectable [44]. A study has shown that the level of BILF2-IgG antibodies in the serum of Burkitt's lymphoma patients is increased [45]. Study has also found that the enhancer on the BILF2 gene loops with the BALF locus and is associated with the expression of the BARF1 gene [46]. However, the function of this gene is not yet clear and requires further research to explore.

The carcinogenic role of EBV is well established, but further research is still needed on its carcinogenic mechanism. Previous studies have shown that EBV-encoded LMP1 can influence the expression of some tumor suppressor genes by targeting the DNMTs and further promote tumor development [9–12]. Recently, Li et al. [47] proposed a possible carcinogenic mechanism for EBNA1. EBNA1 can bind to multiple repeated palindromic DNA sequences in chromosome 11q23 and promote chromosome breakage at this fragile site when the expression of EBNA1 increase. In this study, we described the EBV genomic methylation profiles of several EBV-associated tumors and found differentially methylated genes within them. The differences in methylation modifications may indicate the differences in gene expression levels. Further research on the corresponding expression products may reveal the carcinogenic mechanism of EBV in different tumors.

This study also has some limitations. Firstly, the sample size is limited. Due to the low incidence rate of NKTCL, it is difficult to obtain samples. In order to validate the results more effectively, we will expand the study sample size by extending the sampling period and collaborating with other hospitals. Secondly, there is a large difference in the success rate of capturing sites in different samples. The next step could be to enhance capture success by increasing the number of samples tested or by selectively testing samples with high EBV loads.

In summary, the results of this study suggest that there are differences in EBV methylation profiles among different EBV-associated tumors. Specifically, the significant differences in EBV BILF2 gene methylation in NPC and nasal NKTCL nasopharyngeal brushing samples may serve as an auxiliary diagnostic method. Our work also provides ideas for the application of EBV methylation in the diagnosis of other EBV-related diseases or in other virus-related diseases.

### Supplementary Information

**Additional file 1: Fig. S1.** EBV DNA methylation detection was conducted in methylated and unmethylated standard plasmids. **A** Amplification curves of two standard plasmids under FAM fluorescence channel. **B** Amplification curves of two standard plasmids under HEX fluorescence channel. **Fig. S2.** Scatter plot comparing the methylation levels of each CpG site within 1,44,189 to 1,45,136 bp of the EBV sequence between the four groups. **Fig. S3.** Comparison of methylation levels at gene BILF2 CpG sites obtained by capture sequencing between NPC and nasal NKTCL samples. **A** The methylation levels of BILF2 were compared between NPC and nasal NKTCL nasopharyngeal brushing samples. **B** Scatter plot comparing the methylation levels of each CpG site on the BILF2 between the two groups. **Fig. S4**. Comparison of brushing quality between NPC and nasal NKTCL groups. The quality of samples was evaluated by β-globin detection, and there was no significant difference. **Fig. S5**. The mRNA and protein levels of DNMTs in four types of tumor tissues. **A** The mRNA levels of DNMTs in lung LELC, parotid LELC, EBVaGC and NPC tissues. **B, C** The protein levels of DNMTs in lung LELC, parotid LELC, EBVaGC and NPC tissues. **Table S1.** Clinicopathologic characteristics of the study subjects. **Table S2.** Performance evaluation of EBV DNA methylation detection method. **Table S3**. Primers and probe sequence using in this study.

## Data Availability

The raw sequence data reported in this paper have been deposited in the Genome Sequence Archive [48] of the National Genomics Data Center [49], China National Center for Bioinformation/Beijing Institute of Genomics, Chinese Academy of Sciences that are publicly accessible at https://ngdc.cncb.ac.cn/gsa-human. The accession number is HRA006251.

## Abbreviations

EBV: Epstein–Barr virus
NPC: Nasopharyngeal carcinoma
EBVaGC: EBV-associated gastric cancer
LELC: Lymphoepithelioma-like carcinoma
BL: Burkitt's lymphoma
NHL: Non-Hodgkin lymphoma
HL: Hodgkin's lymphoma
Wp: W promoter
Cp: C promoter
NKTCL: Extranodal NK/T-cell lymphoma
qPCR: Quantitative polymerase chain reaction
qMSP: Quantitative methylation-specific PCR
Ct: Cycle threshold
ROC: Receiver operating characteristic
COV: Cutoff value
AUC: Area under the curve
IM: Infectious mononucleosis
BARTs: BamHI-A rightward transcripts
SNPs: Single nucleotide polymorphisms
