# Supplementary material for: EBV DNA methylation profiles and its application in distinguishing nasopharyngeal carcinoma and nasal NK/T-cell lymphoma
Source: Clin Epigenetics. 2024 Jan 11;16:11. doi: 10.1186/s13148-024-01624-y (PMC10785554; doi:10.1186/s13148-024-01624-y)
Supplement: Supplementary file 1 — Additional file 1: Fig. S1. EBV DNA methylation detection was conducted in methylated and unmethylated standard plasmids. A Amplification curves of two standard plasmids under FAM fluorescence channel. B Amplification curves of two standard plasmids under HEX fluorescence channel. Fig. S2. Scatter plot comparing the methylation levels of each CpG site within 1,44,189 to 1,45,136 bp of the EBV sequence between the four groups. Fig. S3. Comparison of methylation levels at gene BILF2 CpG sites obtained by capture sequencing between NPC and nasal NKTCL samples. A The methylation levels of BILF2 were compared between NPC and nasal NKTCL nasopharyngeal brushing samples. B Scatter plot comparing the methylation levels of each CpG site on the BILF2 between the two groups. Fig. S4. Comparison of brushing quality between NPC and nasal NKTCL groups. The quality of samples was evaluated by β-globin detection, and there was no significant difference. Fig. S5. The mRNA and protein levels of DNMTs in four types of tumor tissues. A The mRNA levels of DNMTs in lung LELC, parotid LELC, EBVaGC and NPC tissues. B, C The protein levels of DNMTs in lung LELC, parotid LELC, EBVaGC and NPC tissues. Table S1. Clinicopathologic characteristics of the study subjects. Table S2. Performance evaluation of EBV DNA methylation detection method. Table S3. Primers and probe sequence using in this study. [file 13148_2024_1624_MOESM1_ESM.docx]

EBV DNA Methylation profiles and its application in distinguishing nasopharyngeal carcinoma and nasal NK/T cell lymphoma

Cao-Li Tang^1,2^, Xi-Zhao Li^1^, Ting Zhou^1^, Chang-Mi Deng^1^, Cheng-Tao Jiang^1^, Yu-Meng Zhang^1,2^, Ying Liao^1^, Tong-Min Wang^1^, Yong-Qiao He^1^, Wen-Qiong Xue^1^, Wei-Hua Jia^1,2,*^ and Xiao-Hui Zheng^1,*^

^1^ State Key Laboratory of Oncology in South China, Guangdong Key Laboratory of Nasopharyngeal Carcinoma Diagnosis and Therapy, Guangdong Provincial Clinical Research Center for Cancer, Sun Yat-sen University Cancer Center, Guangzhou 510060, P. R. China; and ^2^School of Public Health, Sun Yat-sen University, Guangzhou 510080, China

[*] Corresponding Author:

Prof. Xiao-Hui Zheng

Sun Yat-sen University Cancer Center

651 Dongfeng East Road, Guangzhou, Guangdong 510060, China

Tel: 8620 8734 3370; Fax: 8620 8734 3392

Email: zhengxh@sysucc.org.cn

Prof. Wei-Hua Jia

Sun Yat-sen University Cancer Center

651 Dongfeng East Road, Guangzhou, Guangdong 510060, China

Tel: 8620 8734 2327; Fax: 8620 8734 3392

Email: [jiawh@sysucc.org.cn](mailto:jiawh@sysucc.org.cn)

**Figure S1.** EBV DNA methylation detection was conducted in methylated and unmethylated standard plasmids. (A) Amplification curves of two standard plasmids under FAM fluorescence channel. (B) Amplification curves of two standard plasmids under HEX fluorescence channel.


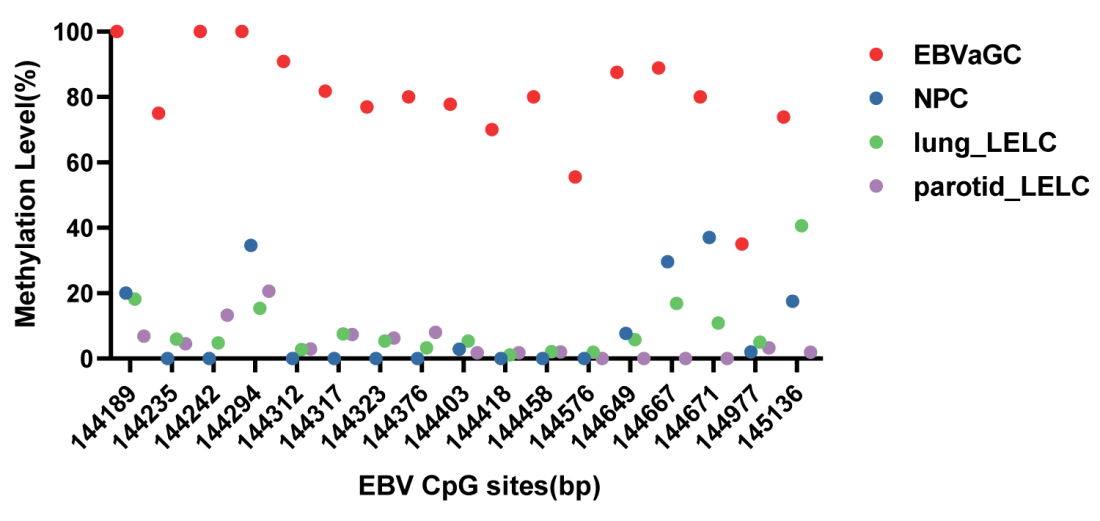


**Figure S2.** Scatter plot comparing the methylation levels of each CpG site within 144,189 to 145,136 bp of the EBV sequence between the four groups.


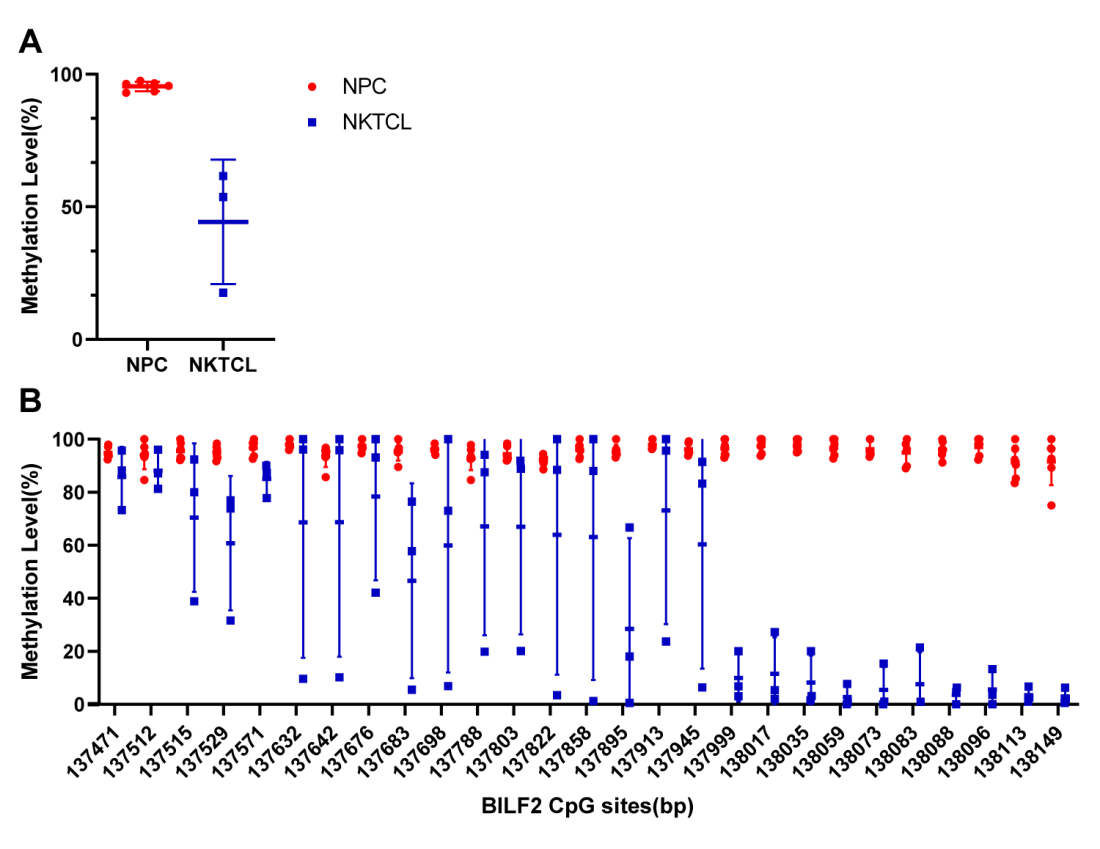


**Figure S3.** Comparison of methylation levels at gene BILF2 CpG sites obtained by capture sequencing between NPC and nasal NKTCL samples. (A) The methylation levels of BILF2 were compared between NPC and nasal NKTCL nasopharyngeal brushing samples. (B) Scatter plot comparing the methylation levels of each CpG site on the BILF2 between the two groups.


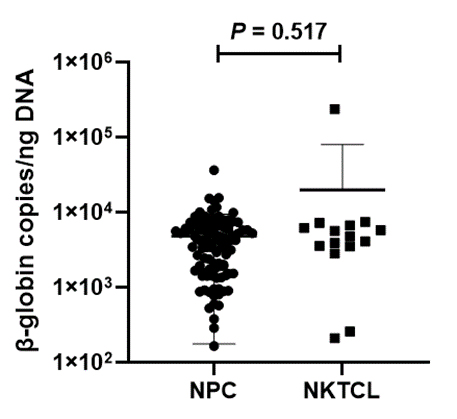


**Figure S4.** Comparison of brushing quality between NPC and nasal NKTCL groups. The quality of samples was evaluated by β-globin detection, and there was no significant difference.


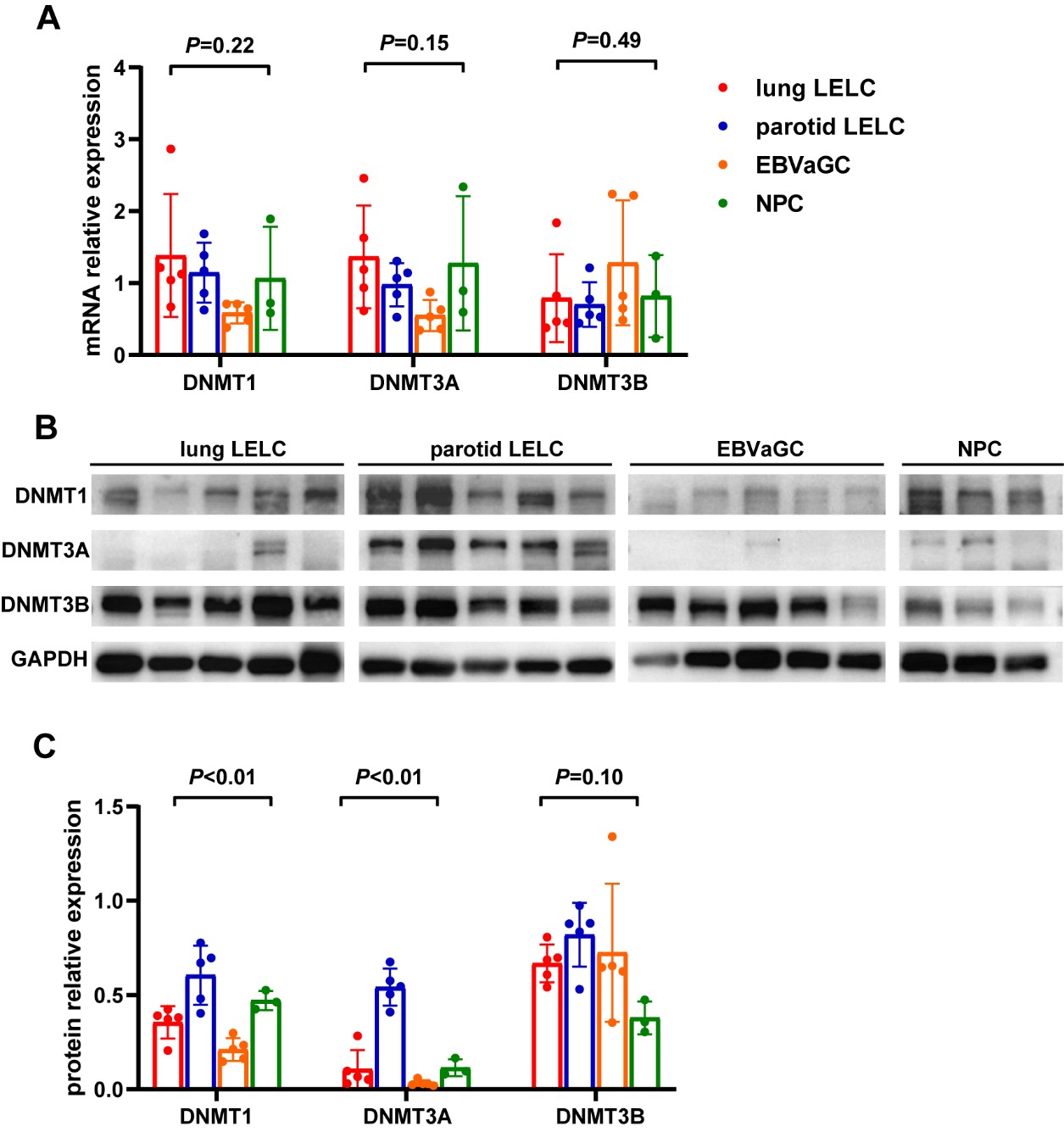


**Figure S5.** The mRNA and protein levels of DNMTs in four types of tumor tissues. (A) The mRNA levels of DNMTs in lung LELC, parotid LELC, EBVaGC and NPC tissues. (B-C) The protein levels of DNMTs in lung LELC, parotid LELC, EBVaGC and NPC tissues.

| **Table S1** Clinicopathologic characteristics of the study subjects | | | | | | | | |
| --- | --- | --- | --- | --- | --- | --- | --- | --- |
| Study phase | Group | Sample type | EBV load^*^ | Stage | | | | |
|  |  |  |  | Ⅰ | Ⅱ | Ⅲ | Ⅳ | - |
| EBV capture sequencing | NPC | tissue | 5.79±0.58 | - | - | 4 | 6 | - |
|  | EBVaGC | tissue | 7.41±0.57 | - | 4 | 5 | 1 | - |
|  | lung LELC | tissue | 7.92±0.35 | 3 | 5 | 1 | 1 | - |
|  | partiod LELC | tissue | 7.67±0.44 | - | - | 1 | 3 | 6 |
|  | NPC | nasopharyngeal brush | 6.00±0.31 | - | - | 2 | 3 | 1 |
|  | NKTCL | nasopharyngeal brush | 4.27±0.48 | - | - | - | 2 | 1 |
| qMSP | NPC | nasopharyngeal brush | 3.26±1.47 | 2 | 6 | 38 | 37 | 20 |
|  | NKTCL | nasopharyngeal brush | 3.83±2.11 | 2 | 4 | - | 3 | 6 |
| *The calculation method for EBV load indicators is log_10_(copy/μl+1) -There is no tumor staging data available | | | | | | | | |

| **Table S2** Performance evaluation of EBV DNA methylation detection method | | | |
| --- | --- | --- | --- |
| Plasmid Type | Concentration (copied/μl) | Ct for Methylated products | Ct for Unmethylated products |
| Methylated | 10^8 | 12.57 | undetected |
|  | 10^7 | 16.91 | undetected |
|  | 10^6 | 21.05 | undetected |
|  | 10^5 | 23.96 | undetected |
|  | 10^4 | 27.61 | undetected |
|  | 10^3 | 31.04 | undetected |
|  | 10^2 | 34.41 | undetected |
| Unmethylated | 10^8 | undetected | 16.05 |
|  | 10^7 | undetected | 20.36 |
|  | 10^6 | undetected | 24.42 |
|  | 10^5 | undetected | 28.07 |
|  | 10^4 | undetected | 31.55 |
|  | 10^3 | undetected | 33.60 |
|  | 10^2 | undetected | 35.51 |

| **Table S3** Primers and probe sequence using in this study | |
| --- | --- |
| Name | Sequence (5' to 3') |
| BamHI-W-F | CCCAACACTCCACCACACC |
| BamHI-W-R | TCTTAGGAGCTGTCCGAGGG |
| BamHI-W-probe | FAM-CACACACTACACACACCCACCCGTCTC-BHQ1 |
| BILF2-F | TGGAAGTAGTTACGGTTAAGG |
| BILF2-R | TCACCGCCCATACAAATACTA |
| BILF2-M-probe | FAM-ATTTTCGGGAGTGTATTTTCGGTTT-BHQ1 |
| BILF2-U-probe | HEX-TATTTTTGGGAGTGTATTTTTGGTTTA-BHQ1 |
| β-globin-F | GTGCACCTGACTCCTGAGGAGA |
| β-globin-F | CCTTGATACCAACCTGCCCAG |
| β-globin-probe | FAM-AAGGTGAACGTGGATGAAGTTGGTGG-BHQ1 |
